# Prevalence of falls and associations with family functioning among community-dwelling older adults in Guangzhou, China

**DOI:** 10.3389/fpubh.2024.1450745

**Published:** 2024-12-12

**Authors:** Si-Yu Sun, Zhi-Wei Wang, Zhi-Li Peng, Le-Xin Yuan, Wen-Hao Yang, Wen-Jian Zhang, Jia-Min Chen, Wei-Quan Lin, Wei Zhu

**Affiliations:** ^1^School of Public Health, Southern Medical University, Guangzhou, China; ^2^Department of 12320 Hotline Management, Center for Disease Control and Prevention of Guangzhou, Guangzhou, China; ^3^Brain Hospital of Guangzhou Medical University, Guangzhou Huiai Hospital, Guangzhou, China; ^4^School of Biomedical Engineering, Guangzhou Medical University, Guangzhou, China; ^5^Department of Basic Public Health, Center for Disease Control and Prevention of Guangzhou, Guangzhou, China; ^6^Department of Science and Education, Center for Disease Control and Prevention of Guangzhou, Guangzhou, China

**Keywords:** falls, older adults, risk factors, family functioning, LASSO regression, association rules

## Abstract

**Introduction:**

Falls are the primary cause of unintentional fatalities among individuals aged 65 and older. Enhancing research on fall prevention among older adults is an urgent priority. Consequently, this study aims to investigate the prevalence and influencing factors of falls among community-dwelling older adults in Guangzhou, China, with a particular emphasis on the impact of family functioning.

**Methods:**

We used a multi-stage stratified cluster random sampling technique to successfully survey 2,399 individuals aged 65 and above across 11 districts in Guangzhou City. Data on sociodemographic characteristics, health and lifestyle factors, and fall incidents were collected through telephone interviews. Chi-square tests, Lasso regression, and logistic regression were utilized to pinpoint fall risk factors. Association rule mining uncovered the relationships between falls and associated variables.

**Results:**

A total of 390 participants reported experiencing falls, the prevalence of falls among older adults was 16.3% (95%CI: 14.82% ~ 17.78%). Logistic regression analysis identified several risk factors for falls among older adults: female [OR = 1.511, 95%CI (1.188–1.922)], age 85 years and older [OR = 2.332, 95%CI (1.447–3.758)], stroke [OR = 1.821, 95%CI (1.038–3.192)], hypoglycemia [OR = 1.639, 95%CI (1.228–2.186)], visual impairment [OR = 1.418, 95%CI (1.097–1.833)], need to be cared for [OR = 1.722, 95%CI (1.339–2.215)], chronic pain [OR = 1.663, 95%CI (1.302–2.124)], and anxiety [OR = 1.725, 95%CI (1.243–2.395)]. In addition, it was shown that a well-functioning family was a protective factor against falls [OR = 0.589, 95%CI (0.44–0.789)].

**Conclusion:**

The prevalence of falls among community-dwelling older adults in Guangzhou City was high, and the influencing factors were complex. It is recommended to develop and implement comprehensive intervention measures for high-risk groups, including those who are females, older adults, and suffer from chronic diseases while paying special attention to the care of family members for older adults.

## Introduction

With the rapid development of the socio-economic and healthcare sectors, population health has improved significantly, and the life expectancy of the residents in different countries has also increased as a whole (1). According to data reported by the National Bureau of Statistics (NBS), by the end of 2022, China’s population of older people aged 65 and above will be close to 210 million, accounting for 14.9% of the total population. China is facing the problem of population aging (2). Aging is associated with changes such as increasing aging of body organs, increased prevalence of chronic diseases, and impaired ability to perform activities of daily living, which makes older adults a high-risk group for falls (3).

Falls were defined as a sudden, involuntary, unintentional change in body position to the same or lower plane than the starting position (4). According to WHO estimates, globally 28–35% of people aged 65 years and older experience at least one fall each year, and the prevalence of falls increases with age (5). In China, the prevalence of falls among older adults is 23.4% (6). The injuries caused by falls among older adults can lead to soft tissue damage, fractures, paralysis, craniocerebral injuries, and even death (7). Data show that falls are the leading cause of injury-related deaths among older adults aged 65 years and older (6, 8). The high prevalence and serious consequences of falls in older adults not only result in functional impairment, disability, or death, leading to a decline in long-term quality of life but also increase the burden on families and society (9). Therefore, falls among older adults are an important public health problem that has attracted widespread attention worldwide.

The factors influencing falls are complicated and the causes of falls may be varied in different countries, regions, and economic backgrounds. The prevention of falls among older adults has been taken up as an important research topic in China and a series of related studies have been carried out. The majority of older adults live in the community for a long time and use the family as the center of their lives. Family is the most basic and important living unit in human society, which provides certain environmental conditions for the healthy development of family members in terms of physical, psychological, and social support. However, the association between falls and older adult family functioning has not been reported. In summary, this study aimed to further explore the prevalence and influencing factors of falls among community-dwelling older adults in Guangzhou, China, focusing on its association with family functioning in particular.

## Methods

### Study design and sampling

This is a cross-sectional study of the prevalence of falls and their association with risk factors among 2,399 participants aged 65 years and older in 2023 in Guangzhou. Structured questionnaires for telephone interviews were adopted, and the interviews were conducted by a group of trained hotline staff and medical students in this study. Social expectation bias and non-response bias are two common biases in telephone interviews. We used measures such as avoiding sensitive questions in the questionnaire and promising confidentiality to the respondents to minimize social expectation bias, and we made multiple contacts with non-respondents to minimize missed interviews.

There are 11 districts in Guangzhou, each of which may differ in terms of economy, culture, and demographics. Consequently, stratified sampling should be used to ensure balanced coverage of the characteristics of each district, randomly select clusters to ensure sampling fairness, and stage-by-stage meticulous sampling to accommodate different strata, while considering intra-cluster and inter-cluster differences and determining a sufficient sample size so that the samples can accurately reflect the overall situation of the city of Guangzhou. Therefore, the researchers were selected based on a multi-stage stratified whole cluster random sampling method. In the first stage, ≥5 community health service centers were randomly selected as monitoring sites in each district of Guangzhou City according to the systematic sampling method of population size ordering; in the second stage, two neighborhood committees/village committees were randomly selected in each of the sampled community service centers; in the third stage, several householders were randomly selected; and in the fourth stage, a whole group of sampling was done among the sampled householders, in which older adults who met the inclusion and exclusion criteria were included as the survey respondents. The inclusion criteria were older adults in the population aged 65 years and older, with clear awareness and strong expression ability. The exclusion criteria were older adults who suffered from severe mental illness during the survey period and were unable to cooperate in completing the questionnaire.

A total of 5,004 older adults were selected as study participants. A total of 2,501 older adults were surveyed excluding death, missing phone numbers, and refusal to answer the phone. After data processing, 102 individuals with missing information were excluded, and 2,399 study participants were finally included.

### Instruments

A self-designed questionnaire on the health status of older adults was used in this study, which was constituted by socio-demographic characteristics, health and lifestyle factors, and the assessment of falls and home functioning.

### Socio-demographic characteristics

These measured the socio-demographic information, including gender, age, area of residence, marital status, education level, and medical insurance.

### Health and lifestyle factors

Lifestyle-related factors included asking investigators about smoking, alcohol consumption, and physical exercise. Health-related factors included chronic diseases (diabetes, hypertension, osteoarthritis, osteoporosis, stroke, heart disease, dyslipidemia, kidney disease, and cancer), use of insulin, hypoglycemic and antihypertensive drugs, the presence of hypoglycemia, orthostatic hypotension, visual impairment, chronic pain, anxiety, and the need to be cared for, sleep quality status, frequency of easy or early awakening, sleep duration and the family functioning.

### Assessment of family functioning

Family functioning was assessed using the Family Care Index Scale in five dimensions: family adaptation, partnership, growth, affection, and resolve (APGAR). For each question, there were three answers to choose from: “often” scored 2 points, “sometimes” scored 1 point, and “almost rarely” scored 0 points. The scores for the five questions were summed to produce a total score, with 7–10 indicating good family functioning and 0–6 indicating family dysfunction.

### Assessment of falls

The assessment of falls was based on the question, Have you fallen in the past 2 years? Falls were defined as a sudden, involuntary, unintentional change in body position to the same or lower plane than the starting position (4). This includes slipping, tripping, fainting, being accidentally bumped or knocked over.

### Ethics statement

Ethical approval for this study was obtained from the Ethics Committee of the Center for Disease Control and Prevention of Guangzhou.

### Statistical analyses

Statistical analyses were performed using R 4.3.2 and Statistical Package for Social Sciences (SPSS), version 25.0 (SPSS Inc., Chicago, IL, United States). Categorical data were described by frequency and percentage. Univariate analysis was performed with the χ^2^ test to explore the effect of each explanatory variable on the prevalence of falls. A binomial logistic regression model was used for the analysis, and significant explanatory variables from the univariate analysis were included in the multivariate logistic regression analysis to identify factors associated with falls. Odds ratios (OR) and 95% confidence intervals (95% CI) were presented. Lasso regression was performed with R software to screen for explanatory variables associated with falls. Association rule algorithms are used to explore the association between falls and related factors. Two-tailed *p* values <0.05 were considered statistically significant in all performed analyses.

## Results

A total of 2,399 older adults aged 65 years and older were investigated in this study in Guangzhou, of whom 390 reported falls, resulting in the prevalence of falls was 16.3% (95% CI: 14.82% ~ 17.78%). Univariate analyses of falls and sociodemographic characteristics, health and lifestyle factors, and family functioning are shown in Tables 1, 2, respectively.

**Table 1:** Prevalence of falls and its association with socio-demographic characteristics among community-dwelling older people in Guangzhou, China.

Variables	Total	No falls	Falls	*χ^2^*	*p* ^c^
Gender (*n*, %)					
Male	1,029 (42.9)	900 (87.5)	129 (12.5)	18.319^a^	<0.001
Female	1,370 (57.1)	1,109 (80.9)	261 (19.1)		
Age groups, years (*n*, %)					
65–74	1781 (74.3)	1,525 (85.6)	256 (14.4)	26.034^a^	<0.001
75–84	519 (21.6)	416 (80.2)	103 (19.8)		
85~	99 (4.1)	68 (68.7)	31 (31.3)		
Area (*n*, %)					
Urban	1,630 (67.9)	1,371 (84.1)	259 (15.9)	0.504^a^	0.478
Rural	769 (32.1)	638 (83.0)	131 (17.0)		
Marital status (*n*, %)					
Married	2,106 (87.8)	1,782 (84.6)	324 (15.4)	9.634^a^	0.002
Others^b^	293 (12.2)	227 (77.5)	66 (22.5)		
Education (*n*, %)					
Middle school and below	1,645 (68.6)	1,382 (84.0)	263 (16.0)	0.278^a^	0.598
High school and above	754 (31.4)	627 (83.2)	127 (16.8)		
Insured (*n*, %)					
No	77 (3.2)	60 (77.9)	17 (22.1)	1.980^a^	0.159
Yes	2,322 (96.8)	1,949 (83.9)	373 (16.1)		

a 0 cells (0.0%) have expected count less than 5.

b Others: unmarried, divorced or widowed.

c Differences between means within each variable, chi-square test analysis for independent samples.

**Table 2:** Prevalence of falls and its association with health and lifestyle factors among community-dwelling older people in Guangzhou, China.

Variables	Total	No falls	Falls	*χ^2^*	*p* ^d^
Cigarette smoking (*n*, %)					
No	1,968 (82.0)	1,626 (82.6)	342 (17.4)	10.116^a^	0.001
Yes	431 (18.0)	383 (88.9)	48 (11.1)		
Alcohol drinking (*n*, %)					
No	1,780 (74.2)	1,485 (83.4)	295 (16.6)	0.507^a^	0.477
Yes	619 (25.8)	524 (84.7)	95 (15.3)		
Physical exercise (*n*, %)					
No	1,859 (77.5)	1,548 (83.3)	311 (16.7)	1.355^a^	0.244
Yes	540 (22.5)	461 (85.4)	79 (14.6)		
Diabetes (*n*, %)					
No	1,923 (80.2)	1,630 (84.8)	293 (15.2)	7.409^a^	0.006
Yes	476 (19.8)	379 (79.6)	97 (20.4)		
Hypertension (*n*, %)					
No	1,183 (49.3)	1,010 (85.4)	173 (14.6)	4.571^a^	0.033
Yes	1,216 (50.7)	999 (82.2)	217 (17.8)		
Osteoarthritis (*n*, %)					
No	1,700 (70.9)	1,457 (85.7)	243 (14.3)	16.508^a^	<0.001
Yes	699 (29.1)	552 (79.0)	147 (21.0)		
Osteoporosis (*n*, %)					
No	1,914 (79.8)	1,629 (85.1)	285 (14.9)	12.986^a^	<0.001
Yes	485 (20.2)	380 (78.4)	105 (21.6)		
Stroke (*n*, %)					
No	2,330 (97.1)	1,962 (84.2)	368 (15.8)	12.744^a^	<0.001
Yes	69 (2.9)	47 (68.1)	22 (31.9)		
Heart disease (*n*, %)					
No	2,104 (87.7)	1,767 (84.0)	337 (16.0)	0.722^a^	0.396
Yes	295 (12.3)	242 (82.0)	53 (18.0)		
Dyslipidemia (*n*, %)					
No	2,076 (86.5)	1,745 (84.1)	331 (15.9)	1.107^a^	0.293
Yes	323 (13.5)	264 (81.7)	59 (18.3)		
Kidney disease (*n*, %)					
No	2,324 (96.9)	1,953 (84.0)	371 (16.0)	4.685^a^	0.030
Yes	75 (3.1)	56 (74.7)	19 (25.3)		
Cancer (*n*, %)					
No	2,387 (99.5)	2,001 (83.8)	386 (16.2)	2.583^a^	0.108
Yes	12 (0.5)	8 (66.7)	4 (33.3)		
Diabetes complications (*n*, %)					
No	2,262 (94.3)	1,907 (84.3)	355 (15.7)	9.212^a^	0.002
Yes	137 (5.7)	102 (74.5)	35 (25.5)		
Receive insulin treatment (*n*, %)					
No	2,336 (97.4)	1,963 (84.0)	373 (16.0)	5.469^a^	0.019
Yes	63 (2.6)	46 (73.0)	17 (27.0)		
Use of hypoglycemic drugs (*n*, %)					
No	1,979 (82.5)	1,675 (84.6)	304 (15.4)	6.658^a^	0.010
Yes	420 (17.5)	334 (79.5)	86 (20.5)		
Use of antihypertensive drugs (*n*, %)					
No	1,260 (52.5)	1,076 (85.4)	184 (14.6)	5.330^a^	0.021
Yes	1,139 (47.5)	933 (81.9)	206 (18.1)		
Hypoglycemia (*n*, %)					
No	2,060 (85.9)	1,759 (85.4)	301 (14.6)	29.981^a^	<0.001
Yes	339 (14.1)	250 (73.7)	89 (26.3)		
Orthostatic hypotension (*n*, %)					
No	1,868 (77.9)	1,592 (85.2)	276 (14.8)	13.608^a^	<0.001
Yes	531 (22.1)	417 (78.5)	114 (21.5)		
Visual impairment (*n*, %)					
No	1,877 (78.2)	1,610 (85.8)	267 (14.2)	26.162^a^	<0.001
Yes	522 (21.8)	399 (76.4)	123 (23.6)		
Need to be cared for (*n*, %)					
No	533 (22.2)	392 (73.5)	141 (26.5)	52.339^a^	<0.001
Yes	1,866 (77.8)	1,617 (86.7)	249 (13.3)		
Chronic pain (*n*, %)					
No	1,100 (45.9)	980 (89.1)	120 (10.9)	42.674^a^	<0.001
Yes	1,299 (54.1)	1,029 (79.2)	270 (20.8)		
Sleep quality (*n*, %)					
Better	1,133 (47.2)	984 (86.8)	149 (13.2)	24.720^a^	<0.001
General	689 (28.7)	578 (83.9)	111 (16.1)		
Worse	577 (24.1)	447 (77.5)	130 (22.5)		
Frequency of easy or early awakening (*n*, %)					
<1 time/week	829 (34.6)	728 (87.8)	101 (12.2)	15.439^a^	<0.001
1–2 times/week	277 (11.5)	226 (81.6)	51 (18.4)		
≥3 times/week	1,293 (53.9)	1,055 (81.6)	238 (18.4)		
Sleep duration (*n*, %)					
<6 h	430 (17.9)	348 (80.9)	82 (19.1)	3.045^a^	0.081
≥6 h	1,969 (82.1)	1,661 (84.4)	308 (15.6)		
Anxiety (*n*, %)					
No	2,167 (90.3)	1,853 (85.5)	314 (14.5)	51.374^a^	<0.001
Yes	232 (9.7)	156 (67.2)	76 (32.8)		
Family care index (*n*, %)					
7–10 scores^b^	2,062 (86.0)	1,758 (85.3)	304 (14.7)	24.708^a^	<0.001
0–6 scores^c^	337 (14.0)	251 (74.5)	86 (25.5)		

a 0 cells (0.0%) have expected count less than 5.

b Indicates a well-functioning family.

c Indicates family dysfunctions.

d Differences between means within each variable, chi-square test analysis for independent samples.

The results of the Chi-square test in Table 1 showed that the prevalence of falls among the respondents showed significant differences in gender, age, and marital status (*p* < 0.05), while there were no significant differences in areas of living, education level, and health insurance (*p* ≥ 0.05). The results of the Chi-square test in Table 2 showed that cigarette smoking, diabetes, hypertension, osteoarthritis, osteoporosis, stroke, kidney disease, diabetes complications, receipt of insulin treatment, use of antihypertensive or hypoglycemic drugs, hypoglycemia, orthostatic hypotension, visual impairment, need to be cared for, chronic pain, sleep quality, frequency of easy or early awakening, anxiety, and family functioning differed significantly in association with the prevalence of falls (*p* < 0.05), while alcohol drinking, physical exercise, heart disease, dyslipidemia, cancer, and sleep duration were not significantly associated with the prevalence of falls (*p* ≥ 0.05).

All statistically significant variables in the univariate analysis were subjected to stepwise logistic regression analysis and the results are summarized in Table 3. The results showed that risk factors for falls in older adults were: female [OR = 1.511, 95% CI (1.188–1.922)], age ≥ 85 years [OR = 2.332, 95% CI (1.447–3.758)], stroke [OR = 1.821, 95% CI (1.038–3.192)], hypoglycemia [OR = 1.639, 95% CI (1.228–2.186)], visual impairment [OR = 1.418, 95% CI (1.097–1.833)], need to be cared for [OR = 1.722, 95% CI (1.339–2.215)], chronic pain [OR = 1.663, 95% CI (1.302–2.124)], and anxiety [OR = 1.725, 95% CI (1.243–2.395)]. In addition, it was shown that a well-functioning family was a protective factor against falls [OR = 0.589, 95% CI (0.44–0.789)].

**Table 3:** Association of falls with socio-demographic and health and lifestyle factors among community-dwelling older people in Guangzhou, China.

Variable	OR^a^ (95% CI)	*p*	OR^b^ (95% CI)	*p*
Gender				
Male	Ref			
Female	1.642 (1.306–2.064)	<0.001	1.511 (1.188–1.922)	0.001
Age groups, years				
65–74	Ref			
75–84	1.475 (1.145–1.9)	0.003	1.327 (1.017–1.73)	0.037
85~	2.716 (1.74–4.237)	<0.001	2.332 (1.447–3.758)	0.001
Stroke				
No	Ref			
Yes	2.496 (1.486–4.191)	0.001	1.821 (1.038–3.192)	0.037
Hypoglycemia				
No	Ref			
Yes	2.08 (1.586–2.728)	<0.001	1.639 (1.228–2.186)	0.001
Visual impairment				
No	Ref			
Yes	1.859 (1.462–2.363)	<0.001	1.418 (1.097–1.833)	0.008
Need to be cared for				
No	Ref			
Yes	2.336 (1.848–2.952)	<0.001	1.722 (1.339–2.215)	<0.001
Chronic pain				
No	Ref			
Yes	2.143 (1.699–2.703)	<0.001	1.663 (1.302–2.124)	<0.001
Anxiety				
No	Ref			
Yes	2.875 (2.132–3.877)	<0.001	1.725 (1.243–2.395)	0.001
Family care index				
0–6 scores^c^	Ref			
7–10 scores^d^	0.505 (0.384–0.663)	<0.001	0.589 (0.44–0.789)	<0.001

95%CI, 95% confidence interval.

a Crude OR.

b Adjusted for all other variables included in the table.

c Indicates family dysfunctions.

d Indicates a well-functioning family.

**Figure 1:**
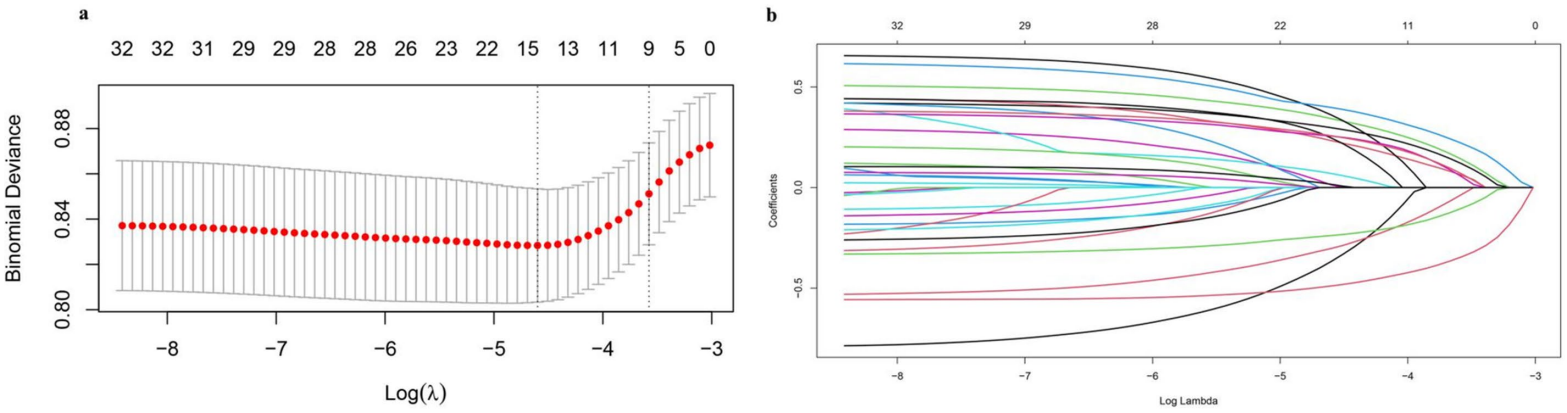
Lasso regression analysis of falls among community-dwelling older adult people in Guangzhou, China. **(a)** LASSO regression cross validation curve plots; **(b)** LASSO regression variable contraction plot.

The variables screened using Lasso regression were generally consistent with the logistic stepwise regression results. Lasso regression achieves variable selection by constructing a penalty function that compresses the coefficients of the variables and makes certain regression coefficients zero. This penalty function is mainly adjusted by the hyperparameter l. The value of l the smallest mean square error (left dashed line in Figure 1A) and the value of 1 one standard error away from the smallest mean square error (right dashed line in Figure 1A) are obtained by 10-fold cross-validation. The latter was chosen as the best l value as it helps to get more streamlined results. According to the optimal hyperparameter l selected in Figure 1A, nine variables with non-zero characteristic coefficients were selected, including gender, age, cigarette smoking, hypoglycemia, visual impairment, need to be cared for, chronic pain, anxiety, and the Family Care Index, and the specific characteristic coefficients are shown in Table 4.

**Table 4:** Lasso variable characterization coefficient.

Variable	Characterization coefficient	Variable	Characterization coefficient	Variable	Characterization coefficient
Gender	0.06120985	Osteoarthritis	0.00000000	Hypoglycemia	0.05386578
Age	0.13993201	Osteoporosis	0.00000000	Orthostatic hypotension	0.00000000
Area	0.00000000	Stroke	0.00000000	Visual impairment	0.06760771
Marital status	0.00000000	Heart disease	0.00000000	Need to be cared for	−0.10282625
Education	0.00000000	Dyslipidemia	0.00000000	Chronic pain	0.21956847
Insured	0.00000000	Kidney disease	0.00000000	Sleep quality	0.00000000
Cigarette smoking	−0.03381937	Cancer	0.00000000	Frequency of easy or early awakening	0.00000000
Alcohol drinking	0.00000000	Diabetes Complications	0.00000000	Sleep duration	0.00000000
Physical exercise	0.00000000	Receive insulin treatment	0.00000000	Anxiety	−0.34406864
Diabetes	0.00000000	Use of hypoglycemic drugs	0.00000000	Family Care Index	0.11797143
Hypertension	0.00000000	Use of antihypertensive drugs	0.00000000		

**Figure 2:**
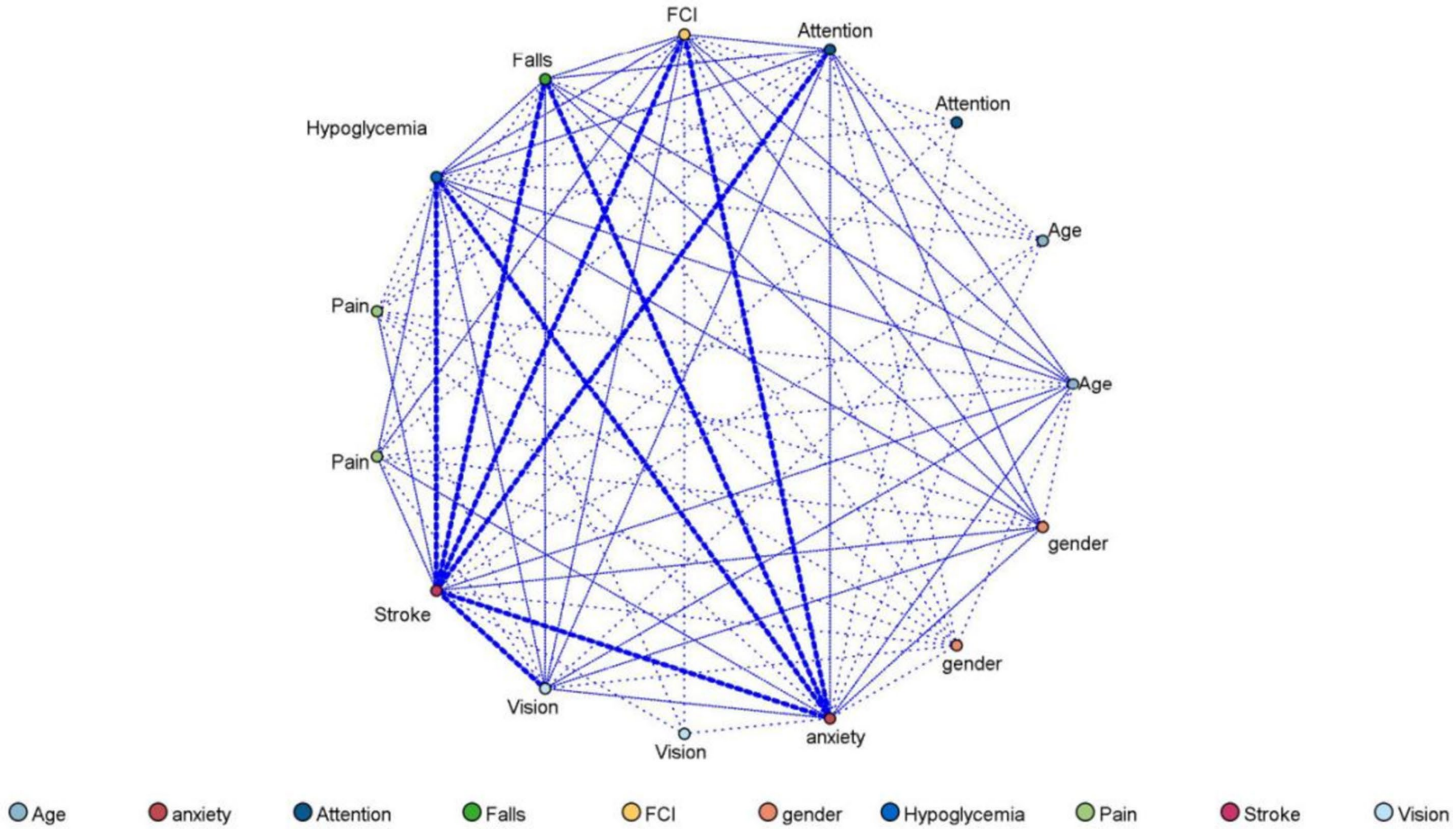
Web diagram of falls among community-dwelling older adults in Guangzhou, China.

The fall patterns of community-dwelling older adults in Guangzhou based on Apriori algorithm association rule mining were demonstrated in Table 5. When there is only one item set on the left-hand side in the association rule, the most common pattern was the model of gender and falls (support: 57.11%, confidence: 19.05). When there were two item sets on the left-hand side, the most common pattern was the model of pain, gender, and falls (support: 34.68%, confidence: 23.08%). Meanwhile, the network diagram of falls among community-dwelling older adults in Guangzhou is presented in Figure 2.

**Table 5:** The pattern of falls based on the Mining Association Rules of Apriori algorithm among community-dwelling older adult people in Guangzhou, China.

Left-hand side		Right-hand side	*n*	Support (%)	Confidence (%)	Lift
With 1 left-hand-side^a^						
1	Hypoglycemia = 1.0	Falls = 1.0	339	14.13	26.25	1.61
2	FCI = 1.0	Falls = 1.0	337	14.05	25.52	1.57
3	Vision = 1.0	Falls = 1.0	522	21.76	23.56	1.45
4	Pain = 1.0	Falls = 1.0	1,299	54.15	20.79	1.28
5	Gender = 1.0	Falls = 1.0	1,370	57.11	19.05	1.17
With 2 left-hand-side^b^						
1	FCI = 1.0 and Pain = 1.0	Falls = 1.0	203	8.46	30.54	1.88
2	Vision = 1.0 and Pain = 1.0	Falls = 1.0	316	13.17	29.75	1.83
3	Vision = 1.0 and gender = 1.0	Falls = 1.0	312	13.01	28.53	1.75
4	FCI = 1.0 and gender = 1.0	Falls = 1.0	184	7.67	28.26	1.74
5	Hypoglycemia = 1.0 and gender = 1.0	Falls = 1.0	221	9.21	28.05	1.73
6	Hypoglycemia = 1.0 and Pain = 1.0	Falls = 1.0	233	9.71	26.18	1.61
7	Hypoglycemia = 1.0 and Attention = 1.0	Falls = 1.0	233	9.71	24.89	1.53
8	Pain = 1.0 and gender = 1.0	Falls = 1.0	832	34.68	23.08	1.42
9	Hypoglycemia = 1.0 and anxiety = 1.0	Falls = 1.0	283	11.80	21.91	1.35
10	FCI = 1.0 and Attention = 1.0	Falls = 1.0	245	10.21	21.63	1.33
11	FCI = 1.0 and anxiety = 1.0	Falls = 1.0	263	10.96	20.15	1.24
12	Vision = 1.0 and anxiety = 1.0	Falls = 1.0	432	18.01	20.14	1.24

a The results of association rules for the relationship between falls and factors with one left-hand-side.

b The results of association rules for the relationship between falls and factors with two left-hand-side.

## Discussion

Our study found that the prevalence of falls was 16.3% among community-dwelling older adults in Guangzhou, China. This was similar to the prevalence of falls among people aged 60 and over in China, Korea, Singapore, and certain parts (Taiwan and Chengdu City) of China (10–14). However, it was lower than the United States (27.5%) (15) and South Africa (20.8%) (16). A systematic review of older adults worldwide showed that the prevalence of falls was 26.5% (17). Differences in the prevalence of falls between regions can be attributed to a variety of factors, including the selection of subjects for the survey, geographic location, sociodemographic differences, and economic level.

The result of the multivariate analysis showed that nine factors (gender, age, stroke, visual impairment, need to be cared for, chronic pain, anxiety, and family functioning) had a relationship with the prevalence of falls. In previous studies, age was a common risk factor for falls, and the prevalence of falls in older adults increased with age (18–20). It may be attributed to degenerative changes in the musculoskeletal system, the decline in gait stability, and the emergence of age-related diseases (such as osteoarthritis, and diabetes mellitus) of older adults with the increase of age, which leads to the increased risk of falls in older adults (21–23). Multiple studies have shown a correlation between gender and the occurrence of falls, with females being more prone to falls than males, which is consistent with our results (24, 25). This may indicate that the differences in falls in older adults stem from gender-related factors. In the years after menopause, women’s muscle strength decreases to some extent faster than men’s (26). At the same time, postmenopausal ovarian dysfunction leads to a decrease in estrogen levels, making women more likely to suffer from osteoporosis. This may explain why men are more prone to falls than men.

In our study, stroke was a risk factor for falls. Our finding is consistent with previous research findings (27). The higher prevalence of falls in the stroke population may be due to an increase in stroke-specific injuries. Muscle weakness or spasticity, sensory deficits, visual field deficits, balance dysfunction, decreased attention, and visuospatial deficits may all increase the risk of falls after a stroke (28–30). In addition, taking psychotropic drugs such as sedative-hypnotics and antidepressants is a risk factor for falls in stroke patients. These drugs directly act on the central nervous system, affecting the patient’s intuition, thinking, behavior, and mood, leading to decreased attention and impaired postural control, which may lead to falls.

Compared with people who had better vision, older adults with visual impairments were more likely to experience falls. This is consistent with previous research findings (31). Perhaps due to insufficient visual input, balance control, and obstacle avoidance abilities are impaired by distance misjudgments and misinterpretation of spatial information. This makes older people more likely to fall.

Our study found that older adults who need to be cared for in their daily lives are more likely to fall. It may be that the pushing and pulling difficulties that occur in daily life lead to losing balance, tripping, and ultimately falling (32, 33). Therefore, older people should be especially careful and try not to push or pull things to reduce the risk of falling. Another important reason is difficulty with bed and chair transfers, also known as sit-to-stand maneuvers, and the inability of older adults to perform this skill can lead to mobility problems, falls, or hospitalization (34). Previous studies have shown that difficulty rising from a chair was associated with decreased lower extremity muscle strength, poor balance, slow reaction time, and an increased risk of falling (35, 36).

In this study, chronic pain was a risk factor for falls in older adults. Previous studies have found that older adults with chronic multisite musculoskeletal pain or more severe generalized pain have reduced mobility and a higher risk of falling compared to older adults without pain (37–39). At the same time, chronic pain affects neuromuscular and motor control, cognitive function, and especially attention, leading to decreased responsiveness to postural perturbations (40–42). These factors may explain the higher prevalence of falls in older adults with chronic pain.

Anxiety is a common mental activity in life that increases the risk of falls in older adults. A study from the United States (43) suggested that fear of falling was correlated with anxiety. In addition, anxiety predicts fear of falling and activity restriction, which may be another way in which anxiety may influence fall risk. Other studies have reported that anxiety could cause a stiffening of the ankle joint, making balancing more difficult when an individual encounters a fall hazard (44). Additionally, anxiety can lead to a decrease in walking speed and stride length, which can also reduce balance. All of these factors combined can result in an increased risk of falls. Measures can be taken to help older adults manage anxiety and reduce the risk of falls due to psychological factors, such as the provision of psychological counseling services.

In our study, a well-functioning family was a protective factor for falls in older adults. First, a well-functioning family enhances the sense of security of older adults. The care and attention of family members can enable older adults to receive timely assistance when they experience mobility or vision problems and reduce the risk of falls due to solitary movement. Family members can prevent falls by reminding, accompanying or providing necessary assistive devices. Secondly, at the psychological level, a well-functioning family provides older adults with the necessary emotional support and reduces their feelings of anxiety and loneliness. This positive mental state helps older adults stay alert and focused, which in turn reduces the risk of falls. In addition, family members can help older adults maintain their physical vitality and balance by encouraging and participating in moderate physical activities, thereby reducing the risk of falls. At the same time, family members can also assist older adults to undergo regular health check-ups for timely identification and treatment of health problems that may increase the risk of falls.

The strength of this study is its population-based sample and analysis. Meanwhile, this study explored the association between family functioning and falls among older adults. However, some limitations of this study need to be considered. First, it is difficult to exhaust all potential risk factors for falls. For example, we did not make a detailed assessment of factors such as hearing, cognitive status, fear of falling, and environment. More detailed and comprehensive research on factors influencing falls in older adults is needed. Secondly, the data were obtained by conducting telephone interviews, so there may be social expectation bias and non-response bias. Third, due to the cross-sectional method of this study, the causal relationship between the factors studied and falls could not be inferred. Therefore, prospective studies were necessary to determine the prevalence and risk factors for falls.

## Conclusion

In summary, people who are female, with advanced age, stroke, vision impairment, need to be cared for, chronic pain, anxiety, and family dysfunction are more susceptible to suffering falls. Identifying modifiable risk factors for falls in older adults is of importance to public health. The risk factors identified in this study provide a theoretical basis for developing fall prevention strategies for older adults.

## Data Availability

The original contributions presented in the study are included in the article/supplementary material, further inquiries can be directed to the corresponding authors.
